# LCI Engineering for Improved Polystyrene Binding: The Impact of Aromatic Amino Acid Substitutions

**DOI:** 10.1021/acsomega.5c06026

**Published:** 2025-09-30

**Authors:** Raghda A. Singab, Shuaiqi Meng, Ulrich Schwaneberg

**Affiliations:** † Lehrstuhl für Biotechnologie, 9165 RWTH Aachen University, Worringerweg 3, Aachen 52074, Germany; ‡ DWI-Leibniz Institut für Interaktive Materialien, Forckenbeckstrasse 50, Aachen 52074, Germany; § Microbiology and Immunology Department, Faculty of Pharmacy, Ain Shams University, African Union Organization St., Abbassia, Cairo 11566, Egypt

## Abstract

Polystyrene (PS) is a widely used synthetic polymer with applications in biosensing, medical devices, and packaging. PS often requires surface modifications to enhance biocompatibility, adhesion, and chemical functionality. Material-binding peptides (MBPs) provide a biobased and scalable approach for PS functionalization. Therefore, optimizing their binding properties can ensure stable and efficient binding under industrial application conditions. In this study, we systematically explored how aromatic amino acid substitutions (His, Phe, Trp, and Tyr) affect the PS binding ability of the MBP named liquid chromatography peak I (LCI). A total of 178 aromatic amino acid substitutions were evaluated across all 47 positions of LCI, resulting in the identification of 56 substitutions across 32 positions that improved the PS binding. Among these, the LCI-L4H variant showed the most impoved binding to PS and was further biophysically characterized to determine the surface coverage by surface plasmon resonance (SPR). SPR analysis showed that L4H increased the coating density from 7.90 to 9.18 pmol/cm^2^ (5.52 × 10^12^ molecules/cm^2^), which corresponds to surface coverage of approximately 82%. Molecular dynamics (MD) simulations revealed that the LCI-L4H variant is more compact in size and interacts more frequently through π–π interactions with PS. The high surface coverage and the diversity of the provided functional groups of LCI make the MBP-binding coating a promising alternative to chemical or physical methodologies used in PS functionalization.

## Graphical abstract

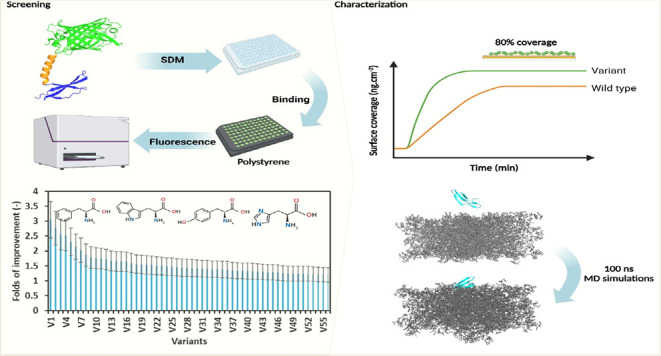

## 1 Introduction

Polystyrene (PS) is a widely used bulk polymer, with a production of 15.44 million tons in 2022,^1^ due to its favorable properties such as lightweight, low cost, ease of processing, and is often used in thermal and electrical insulation.^2–7^ Common products with PS and PS-blends can therefore be found in packaging, construction, electronics, and medicine.^2,3,5,8–15^ In most applications, PS materials are often chemically modified to enhance wettability, adhesion, and printability.^7,16^ Common functionalization techniques can be divided into chemical, physical, and more recently protein- or peptide-based methodologies.^17^ Chemical and physical methods are commonly used in various industrial applications.^7,18–20^ Plasma treatment is, for example, utilized in approximately 80% of semiconductor manufacturing processes.^21^

Chemical surface modifications comprise oxidation, reduction, hydrolysis, and aminolysis,^22^ that introduce functional groups such as hydroxyl-(−OH),^23,24^ carbonyl-(−C=O),^24^ carboxyl-(−COOH),^23–25^ sulfonic-(−SO_3_H),^26,27^ and amino groups (−NH_2_).^23^ The latter modifications are applied by immersing the PS into chemical solutions (dip coating) or by spray coatings.^22^ Depending on the method and treatment conditions, the reported density of functional groups (−OH, −COOH) on a PS surface can reach 1.93 × 10^14^ groups/cm^2^, covering approximately 25% of the surface.^20,25,26^

Physical surface modifications are commonly achieved through plasma treatment.^16,22,28^ Other techniques include corona discharge, flame treatment, and radiation (laser, UV, and γ).^22,29^ Plasma treatment uses gases, such as air,^30^ argon (Ar),^7,31^ nitrogen (N_2_),^32^ oxygen­(O_2_),^7^ carbon dioxide (CO_2_),^18^ and ammonia (NH_3_), depending on intended functionalization.^33^ By applying energy, such as radio frequency, under low pressure, the gas ionizes and forms reactive species, such as radicals and excited molecules.^22^ For example, oxygen plasma produces reactive species such as hydroxyl radicals (OH^•^) and atomic oxygen (O),^34^ while nitrogen plasma generates species such as excited nitrogen molecules (N_2_)* and atomic nitrogen (N).^35^ These reactive species interact with the polymer surface and form surface-bound radicals, such as carbon-centered radicals (^•^C) and oxygen-bound radicals (^•^C–O);^36,37^ with lifetimes ranging from microseconds to milliseconds. Radicals facilitate the introduction of functional groups like amine (−NH_2_), hydroxyl (−OH), and carboxyl (−COOH) groups.^38^ Plasma treatment was reported to achieve functional group densities (−NH_2_, −COOH) of up to 4.5 × 10^14^ groups/cm^2^ on PS, which corresponds to 747.26 pmol/cm^2^. This range of functional group density covers up to 58% of the surface.^39,40^ The stability of radicals generated during plasma treatment poses a main challenge for scale-up, as reactivity diminishes over time.^21^ In plasma-treated polymers, hydrophobic recovery occurs due to surface reorganization and functional group loss, gradually restoring hydrophobicity.^16,29,41^

Material binding peptides (MBPs) are a promising alternative to the standard chemical and physical surface functionalization processes. MBPs allow the introduction of single and multiple functional groups within one peptide comprising sulfhydryl-, amino-, hydroxyl-, and carboxyl groups;^42^ in addition, secondary structures such as ß-strands or α-helices enable a 3-D positioning of functional groups to even control self-assembly processes.^43,44^ MBPs can be classified into naturally occurring binding peptides (nMBPs), such as carbohydrate-binding modules (CBMs), and man-made or engineered MBPs (eMBPs), such as man-made polymer binding peptides identified, for instance, in phage-display libraries (usually short peptides with <16 aas).^44^ nMBPs and eMBPs have been reported to bind to a variety of materials,^45^ including natural polymers,^46,47^ man-made polymers,^48,49^ ceramics,^43^ metals,^50^ and natural surfaces, such as hair,^51^ teeth,^51^ or plant leaves.^52,53^ Specifically, the MBPs have been proven to bind to various plastics, such as PS, polyethylene terephthalate (PET), and polypropylene (PP).^42,43,54–57^

MBPs can be regarded as an energy- and resource-efficient coating method for biocompatible surface functionalization. MBP-based surface functionalization can be achieved from aqueous solutions at room temperature without requiring harsh conditions such as elevated temperatures or strong acids or bases.^42,58,59^ Although peptides are perceived as cost-intensive, their high surface affinity and functional density allow coatings with minimal quantities. For example, the MBP LCI formed a dense monolayer on PP, with 1-g LCI sufficient to cover over 600 m^2^ of PP-surface. Furthermore, scalable coating processes such as dip and spray coating have successfully been employed for MBPs, allowing high-density surface coverage and large-scale coating at a cost of less than 1 euro cent per m^2^.^42,44,53,60^ Interestingly, a surface coverage of up to >90% within 20 s was reported on carbon nanotubes.^61,62^ This translates to low material cost per area, comparable to or even lower than some chemical functionalization approaches, especially when factoring in the avoidance of harsh solvents, waste generation, and postprocessing. Furthermore, unlike plasma treatment, peptides offer the unique ability to decorate surfaces with biologically functional moieties (e.g., enzymes or targeting motifs), under mild aqueous conditions, with high specificity and minimal environmental burden.^58,63^ MBPs like PB-TUP have been used to immobilize functional peptides on PS surfaces for biosensor and diagnostic applications, validating their utility in polymer functionalization.^45,64^ This enables advanced applications such as biosensing and bioconjugation that are not feasible with conventional treatments. Hence, MBP coatings are not only a complementary alternative but also uniquely suited for functionalizing PS in advanced material applications. With developed directed evolution methodologies,^65–69^ binding properties of MBPs, such as binding strength in the presence of surfactants^54,69^ and material-specific binding,^62^ can be improved and tailored to application demands. As a protein engineering strategy, the KnowVolution process proved to successfully improve the material-specific binding of polylactic acid over PP by 2.3-fold and PS by 2.0-fold for the Cg-Def MB.^62,70^

Recycling mixed plastic is challenging and requires that employed catalysts can material-specifically target a specific polymer within a polymer blend. Inspired by cellulases in nature, which utilize CBM to recognize specific carbohydrate polymers, the MBP TA2 was fused with the cutinase Tcur1278. The fusion enhanced an efficient degradation of the Impranil polymer with up to 6.6-fold enhanced depolymerization.^71^ Multiplexed detection with three Alexa-fluorophores and sorting of PS-nanoparticles by flow cytometer was successfully achieved with the conjugates of the MBP LCI to Alexa-fluorophores.^72^

PS interacts primarily through hydrophobic interactions, π–π stacking, and van der Waals forces.^73–76^ PS dissolves in nonpolar solvents such as benzene and toluene.^74,77^ The dominant role of aromatic residues in PS binding was first proposed in 1995, based on the analysis of peptides identified by phage display.^78^ The first reported binding motif was WXXW (where W is Tryptophan and X represents any amino acid).^78^ In 1996, additional motifs such as the WXXWXXXW motif^79^ and the WHXW motif^80^ (where H is Histidine) were identified, and later studies confirmed the prominent role of aromatic amino acids.^64,81–83^

In this work, we used the peptide LCI, a 47-amino acid MBP with a four-stranded antiparallel β-sheet structure, to comprehensively investigate the influence of the aromatic amino acid substitutions (including Trp, Tyr, Phe, and His) on PS binding. Site-directed mutagenesis (SDM) was performed at all positions in LCI, generating a total of 178 variants to systemically evaluate the contribution of individual residues. A fluorescence-based assay was then used to evaluate the binding of each variant, and a statistical analysis was conducted to determine amino acid preferences and structural distribution patterns. Key variants exhibiting improved PS binding were identified, with the most improved variant (LCI-L4H) subjected to surface plasmon resonance (SPR) for detailed binding characterization. A single substitution at position L4 could increase the surface coverage from 7.90 to 9.18 pmol/cm^2^. Furthermore, molecular dynamics (MD) simulations were conducted to analyze the changes in binding modes, providing deeper insights into the impact of these substitutions. The simulations revealed that the LCI-L4H variant was more flexible than LCI-WT, enabling it to better adapt to the PS surface and present more aromatic residues for surface interactions.

## 2 Results and Discussion

The results section is divided into three parts. First, the LCI aromatic amino acid library was screened for PS binding to identify variants with improved binding properties, followed by a statistical analysis to evaluate their amino acid preferences and structural distribution. Second, the variant LCI-L4H, which showed the highest improvement in PS binding, was purified and characterized by SPR to determine the surface coverage. Finally, MD simulations were performed to investigate the binding interactions between the LCI and the amorphous PS surface.

### 2.1 Screening of eGFP-LCI Aromatic Library for PS Binding

A screening system was first developed and validated in 96-well PS MTPs through fluorescent measurements based on the eGFP-LCI fusion protein (Figure S1, S2). eGFP-LCI was expressed in *Escherichia coli* BL21 (DE3) gold, and clarified cell lysate in 50 mM Tris buffer (pH 8) was used for assay optimization (5 −50 μL; see Figure S3). When less than 15 μL of eGFP-LCI cell lysate was used, fluorescence detection was volume-dependent, which indicates that the well surface was not saturated. In order to eliminate false positives arising from enhanced eGFP-LCI expression, 30 μL of lysate was used in the library screening. To evaluate the reproducibility of the screening system, a binding assay was performed using an MTP containing eGFP-LCI WT, which yielded a true coefficient of variation (CV) of 18.8% (Figure S4). Screening systems with CV below 20% have been routinely and successfully used in protein engineering campaigns.^62,84^

The binding of eGFP-LCI (178 variants) was tested on PS black MTPs in duplicates, with the initial fluorescence levels maintained above 20,000 (gain 1000; Table S1). The threshold for an improved variant was set at 1 + σ, where σ = 0.19. After the initial screening, 65 variants with improved PS binding were selected for rescreening in five replicates. After rescreening, 56 of these variants showed improved PS binding compared to that of the WT (Figure 1).

**Figure 1:**
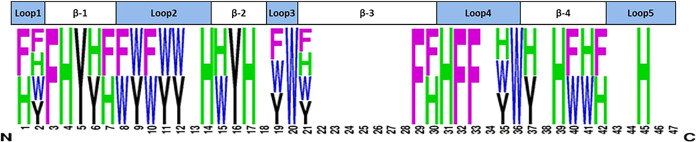
Sequence logo of LCI variants with improved PS binding (generated using WebLogo; weblogo.berkeley.edu), illustrating positions and beneficial amino acid substitutions within the LCI that result in improved PS binding. Interestingly, all four aromatic amino acids seem to contribute equally since no specific amino acid occurs significantly more frequently than the other three.

As shown in Figure 1, beneficial LCI variants with enhanced PS binding were distributed across 32 positions with occurrences ranging from 17 to 11 exchanges (17 × H, 14 × F/14 × W, 11 × Y). Interestingly, H occurred six times as the only beneficial substitution compared to 4 × F, 2 × W, and 2 × Y. At positions 2 and 21, all four aromatic amino acids contributed to the improved PS binding.

LCI is composed of 4 antiparallel β-sheets, and the contribution of different secondary structure elements to improved PS binding was analyzed based on the number of beneficial substitutions. Among the β-sheets, 27 substitutions were observed (9 × β-4, 7 × β-1/7 × β-3, 4 × β-2), while the loops contributed 29 (11 × Loop-2, 6 × Loop-1, 7 × Loop-4, 4 × Loop-3, and 1 × Loop-5). Interestingly, the beneficial variants were distributed across multiple positions of the peptide, suggesting that both loops and β-sheets contribute comparably to the overall binding improvement and that multiple binding modes may be involved. Moreover, the observed substitutions may not only strengthen direct interactions with the PS surface but also influence the peptide’s conformation, indirectly improving its binding efficiency.

### 2.2 Binding Characterization of Variants with Improved PS Binding

All 56 variants with improved PS binding were purified. After purification, their binding was evaluated using PS MTPs. Among these, five variants demonstrated a significant improvement in PS binding compared to WT (1.2- to 1.4-fold; *P*-value <0.05, two-tailed unpaired *t* test; Figure 2). The top variant L4H was selected for further characterization by SPR to quantify the surface coverage.

**Figure 2:**
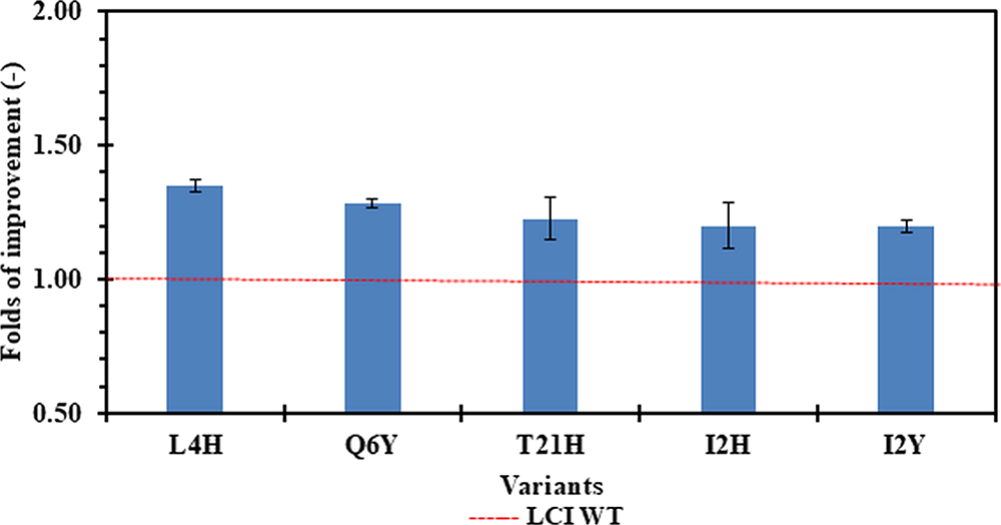
Purified LCI variants with improved PS binding. The bar chart shows the fold improvement in PS binding for purified eGFP-LCI variants relative to the eGFP-LCI-WT calculated by fluorescence ratio eGFP-LCI variant vs eGFP-LCI-WT (excitation = 485 nm, emission = 520 nm). The red dashed line represents the baseline for the LCI WT at a fold improvement of 1.0. Error bars represent the standard deviation across 4 replicates.

A concentration range from 50 to 2000 nM of eGFP-LCI WT and eGFP-LCI L4H was tested on PS-coated SPR chips to analyze binding interactions (Figure 3). Additionally, eGFP alone was tested at a concentration of 500 nM and compared to both the eGFP-LCI WT and eGFP-LCI L4H variant. As shown in Figure 3a, eGFP alone was washed off after 40 min, confirming that binding is mediated through the LCI peptide. For the eGFP-LCI WT, the maximum surface coverage was determined to be 286.2 ± 22.45 ng/cm^2^, corresponding to 7.90 ± 0.61 pmol/cm^2^ based on the protein’s molecular weight (36.21 kDa). In contrast, the eGFP-LCI L4H variant showed improved binding, with a surface coverage of 332.7 ± 12.6 ng/cm^2^, equivalent to 9.18 ± 0.34 pmol/cm^2^ based on its molecular weight (36.24 kDa). This represents a 1.16-fold increase in surface coverage compared with the eGFP-LCI WT (Figure 3b). The molecular surface coverage for LCI-WT is 4.75 × 10^12^ molecules/cm^2^, and for the LCI-L4H variant, it is 5.52 × 10^12^ molecules/cm^2^. To estimate the number of LCI molecules covering the PS surface per unit area, the LCI molecule was approximated to have a rectangular shape. The dimensions of LCI were determined using the PyMOL script Draw_Protein_Dimensions, yielding measurements of 40 × 20 × 20 Å. The LCI molecules were arranged with a spacing of 10 Å between them. Based on this model, the theoretical maximum surface density of LCI is 6.67 × 10^12^ molecules/cm^2^, with the LCI-WT and LCI-L4H variants achieving 71.2% and up to 82.8% coverage, respectively.

**Figure 3:**
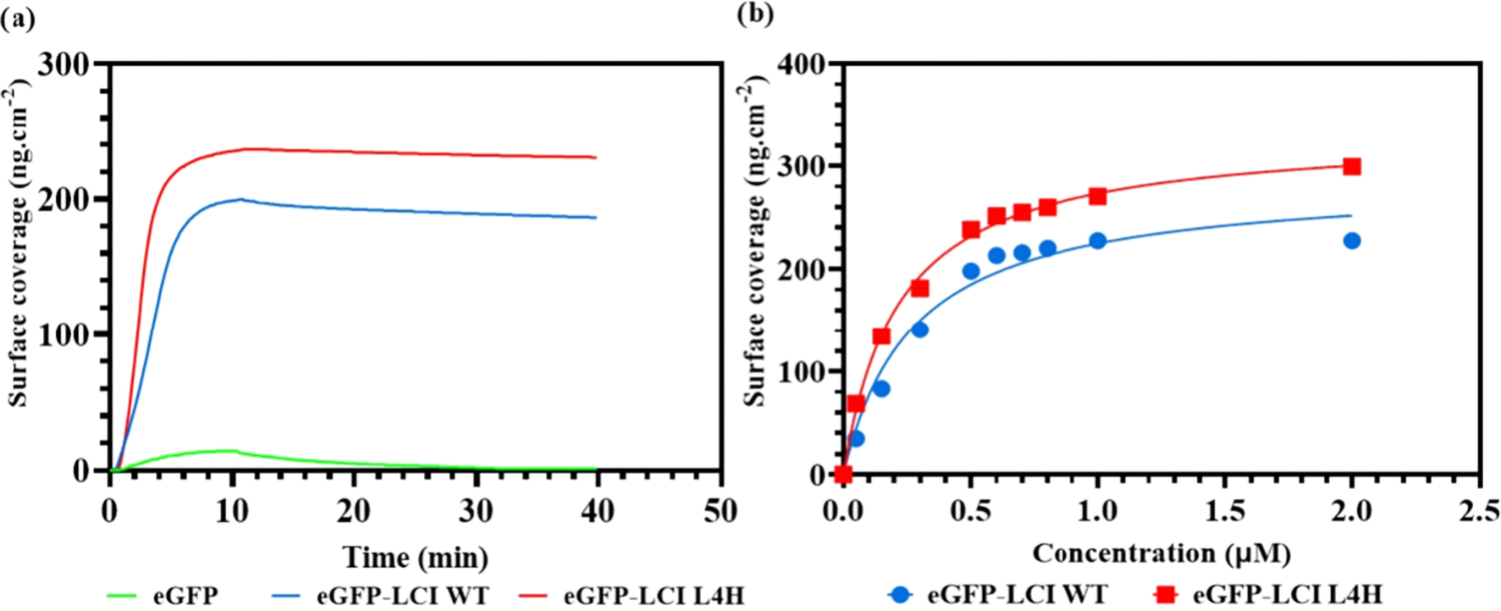
(a) Comparison of surface coverages of the eGFP-LCI WT, eGFP-LCI L4H variant, and eGFP alone at a concentration of 500 nM. (b) Binding curve for eGFP-LCI WT and eGFP-LCI L4H on PS showing surface coverage as a function of concentration.

### 2.3 MD Simulations of LCI-WT and LCI-L4H Binding to PS

The LCI-WT and LCI-L4H were simulated for 100 ns to monitor their binding modes and any changes in secondary structure throughout the simulation. The simulations were conducted under constant temperature and pressure. Secondary structure alterations were tracked over time and visualized using VMD, as shown in Figure S7. A series of analyses were conducted to compare the LCI-L4H variant with the LCI-WT, including root-mean-square fluctuation (RMSF), root-mean-square deviation (RMSD), and radius of gyration (Rg) (Figure S8). Additionally, contact frequency maps were generated to compare the interactions between the peptides and the PS surface.

The Rg analysis showed that the LCI-L4H variant adopts a more compact structure when bound to PS, which may enhance its interaction with the polymer. RMSD analysis revealed that both LCI-WT and LCI-L4H reached equilibrium after approximately 20 ns of simulation. RMSF analysis highlighted that LCI-L4H showed increased flexibility, especially in the N-terminal region (residues 1–23), compared to LCI-WT. This increased flexibility could contribute to improved contact with the PS surface.

Furthermore, the contact frequency between the peptide residues and the polymer surface during the MD simulation was analyzed for both the LCI-WT and the LCI-L4H variant (Figure 4). All simulations were performed in triplicate. The contact patterns were not entirely consistent across all runs (Figure S9). For instance, loop-2 in LCI-WT interacted with the PS surface in one simulation but not in the other two. However, the primary contact regions in both the LCI-WT and the LCI-L4H variant remained consistent across all simulations. As shown in the contact map (Figure 4), residues A1-V5 and I24–E42 in LCI-WT exhibit stable interactions with the PS surface, suggesting that these regions contribute to the peptide’s interaction with PS. In comparison, the LCI-L4H variant showed similar but more extensive contact regions, particularly within residues V5–S15. This suggests that the L4H substitution may contribute to binding indirectly by influencing the orientation of adjacent residues, promoting closer and more frequent interactions with the PS surface. These findings indicate that the LCI-L4H variant exhibits improved surface interactions, which underlie the improved binding and surface coverage observed experimentally.

**Figure 4:**
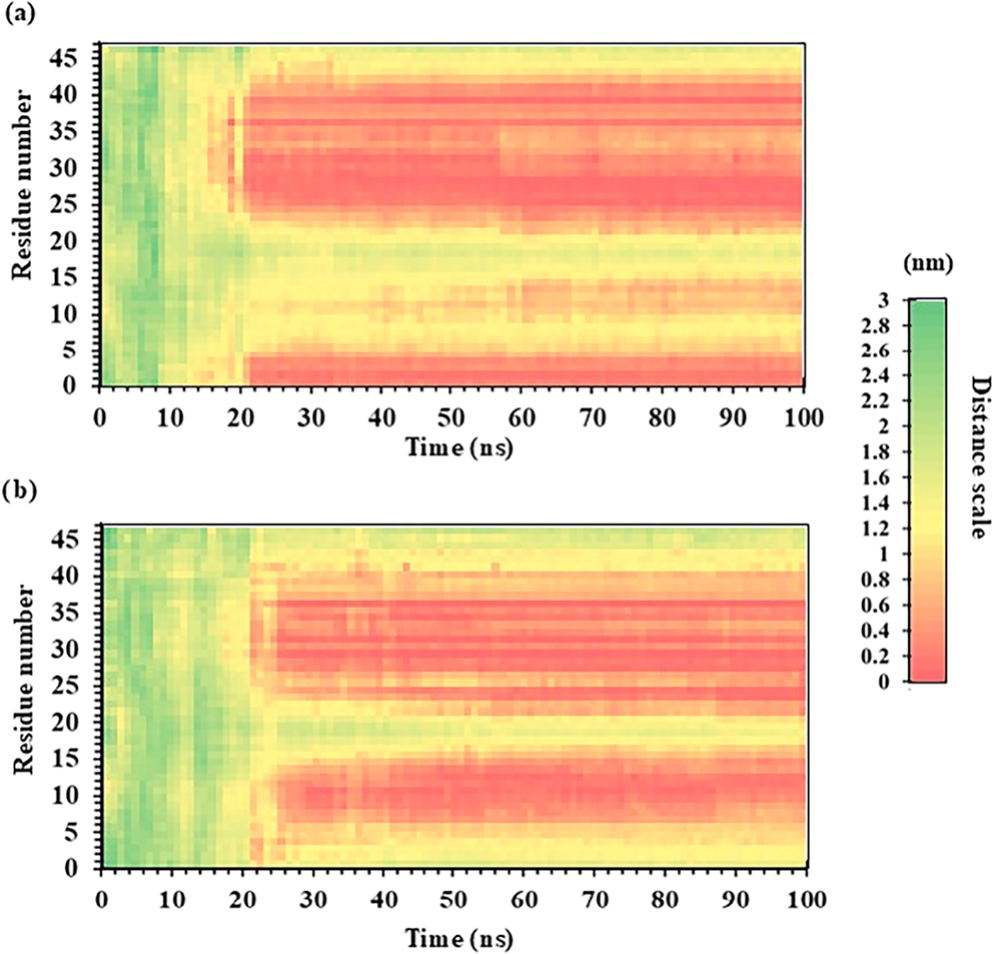
Binding contact frequency for LCI-WT and LCI-L4H variants over 100 ns MD simulations. The heatmaps illustrate the binding contact frequency between PS and the LCI-WT or LCI-L4H variant over a 100 ns MD simulation. The *y*-axis represents residue numbers, while the *x*-axis shows simulation time. Data represents the average of three independent simulation runs. The color scale bar on the right indicates the distance between residues and the PS surface (in nm), with red indicating closer contact (more frequent interactions) and green indicating less frequent. (a) The LCI-WT shows close contact between residues 1–5 and around 24–42, with red regions indicating close and frequent binding interactions. (b) The LCI-L4H variant demonstrates more consistent and frequent close contacts across several residues, particularly in the N-terminal region.

We extracted the binding conformations of the LCI-L4H variant and LCI-WT (Figure 5). A key difference is that, in the LCI-WT, only β-sheets 3 and 4 interact with the PS surface. In contrast, the LCI-L4H variant is positioned closer to the surface and exhibits a larger contact area. Specifically, in the LCI-L4H variant, more aromatic amino acids (F12, F25, Y29, Y30, and W37) interact with the PS surface. In the LCI-WT, only Y29, Y30, and W37 are close enough to the PS surface to form π–π interactions. Across the three simulation runs, the variant presented more aromatic amino acids for surface interactions. Small peptides are highly dynamic, and local substitutions can affect molecular interactions within the peptide.^85^ In the LCI-L4H variant, the larger and aromatic side chain likely disrupts local β-sheet hydrogen bonding, leading to a reorganized conformation that promotes more extensive contact with the surface. This was further confirmed through performing circular dichroism (CD) spectroscopy for eGFP-LCI WT and eGFP-LCI L4H, which showed a slight decrease in the peak intensity for β-sheets, indicating slight destabilization in the β-sheets in the case of the variant (Figure S10). These findings highlight the importance of the peptide conformation and the role of aromatic amino acids in improving PS binding.

**Figure 5:**
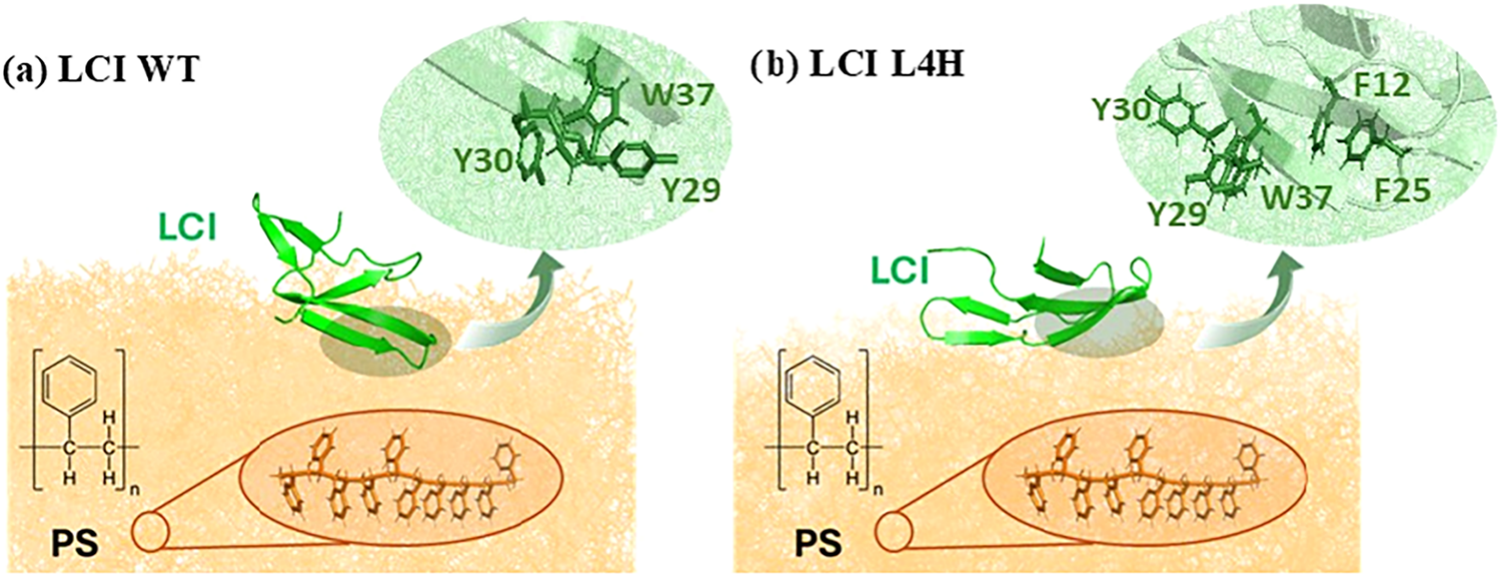
Binding poses of LCI-WT (a) and the LCI-L4H variant (b) on the PS surface. The zoomed-in view showed the aromatic amino acids potentially interacting with the PS surface.

### 2.4 Binding Free Energy

MM/PBSA calculations were performed over three independent simulation runs to estimate the binding free energy of the LCI-WT and the LCI-L4H variant to the PS surface. As shown in Table 1, the LCI-L4H variant displayed a slightly more favorable binding energy (−315.1 ± 23.5 kcal/mol) compared to the LCI-WT (−310.1 ± 17.5 kcal/mol). Despite overlapping standard deviations, the results suggest a trend toward improved surface interaction in the variant, consistent with the proposed role of aromatic substitutions in enhancing PS affinity.

**Table 1:** Binding Free Energy (Δ*G*) of LCI-WT and LCI-L4H Variants to the PS Calculated Using MM/PBSA Across Three Independent Molecular Dynamics Runs^a^

**peptide**	**binding free energy** (kcal/mol)
**LCI-WT**	–310.07 ± 17.47
**LCI-L4H**	–315.09 ± 23.49

a Values are reported in kcal/mol.

## 3 Conclusions

The systematic study of the four aromatic residues His, Trp, Tyr, and Phe at each LCI position (178 variants in total) proved that PS binding and surface coverage of eGFP-LCI fusion proteins can be improved through all four aromatic amino acids. The MBP LCI improved the surface coverage compared to eGFP, while the LCI-L4H variant increased the surface coverage from 7.90 to 9.18 pmol/cm^2^ by a single substitution, achieving approximately 82% surface coverage. Interestingly, a similar number of beneficial substitutions can be found in loops (29) and β-sheets (27) despite the LCI consisting of 22 residues in loops and 25 residues in β-sheets. Being able to tune binding strength and surface coverage to PS through aromatic interactions is a protein design principle that can likely be extended to other natural and man-made polymers with aromatic building blocks, such as PET, polycarbonate, and poly­(3,4-ethylenedioxythiophene) polystyrene sulfonate (PEDOT:PSS). MBPs can provide multiple and different functionalities, such as sulfhydryl- or amino groups on a single MBP, which offers, in combination with tuning binding strength through aromatic interactions, applications in biosensors, (nano/micro-) plastic detection, and sustainable packaging solutions.

## 4 Experimental Section

### 4.1 Materials

All chemicals were purchased from AppliChem GmbH (Darmstadt, Germany), Carl Roth GmbH (Karlsruhe, Germany), Fluka (Ulm, Germany), Macherey-Nagel (Düren, Germany), or Sigma-Aldrich Corp. (St. Louis, MO and Deisenhofen, Germany), unless specified. Salt-free oligonucleotides were obtained from Eurofins Scientific SE (Ebersberg, Germany) and Enzymes from New England Biolabs GmbH (Frankfurt am Main, Germany). Plasmid extraction and polymerase chain reaction (PCR) purification kits were purchased from Macherey-Nagel GmbH & Co. KG (Düren, Germany) and Qiagen GmbH (Hilden, Germany). Black PS MTPs were obtained from Greiner Bio-One GmbH (Frickenhausen, Germany). The plasmid pET28a­(+) (Novagen, Darmstadt, Germany) was used, and the *E. coli* strain BL21-Gold (DE3) (Agilent Technologies, Santa Clara, CA) was used for protein expression.

### 4.2 Library Generation

Aromatic amino acid libraries were generated using SDM. In the LCI (sequence shown in Figure S11), 47 positions were individually substituted with His (p*K*_a_ = 6), Tyr, Trp, or Phe, resulting in a total of 178 variants. The SDM protocol followed the conditions previously outlined.^54^ After amplification, the parental DNA was digested with 20 U of DpnI for 16 h at 37 °C, purified using a PCR cleanup gel extraction kit, and transformed into *E. coli* BL21-Gold (DE3) for expression.

### 4.3 Library Screening

During the screening process, the LCI variants were cultured and expressed in 96-well plates to facilitate a high-throughput analysis. Expression was performed as previously outlined.^42^ For cell lysis, the *E. coli* BL21 (DE3) gold pellets were resuspended in lysozyme solution (150 μL; 1.5 mg/mL in 50 mM Tris/HCl buffer, pH 8.0) and incubated at 37 °C, 900 rpm, and 70% humidity for 1 h. Following lysis, the mixture was centrifuged at 3200*g* for 30 min at 4 °C (Eppendorf centrifuge 5810 R).

Screening of the eGFP-LCI library was performed using 96-well black PS MTPs. For each well, 30 μL of eGFP-LCI cell lysate was mixed with 70 μL of Tris-HCl buffer (pH 8.0, 50 mM) and incubated on an MTP shaker (ELMI DTS-4, ELMI-Tech, Riga, Latvia) at room temperature for 10 min at 600 rpm. After incubation, wells were washed five times with 100 μL of Tris-HCl buffer (pH 8.0, 50 mM) for 5 min at room temperature and 600 rpm. After removal of the liquid, the residual fluorescence from the bound eGFP-LCI was measured directly on the well surface using a 96-well MTP reader (CLARIOstar) with excitation at 485 nm, emission at 520 nm, and a gain setting of 1400, with 35 reads per well. eGFP alone was used as a negative control to exclude nonspecific binding. The improvement of PS binding was calculated by following eq 1.

1 $$\mathrm{improvement}\;\mathrm{of}\;\mathrm{PS}\;\mathrm{binding}=\frac{\left(\mathrm{FI}\;\mathrm{of}\;V-\mathrm{FI}\;\mathrm{of}\;\mathrm{eGFP}\right)}{\left(\mathrm{FI}\;\mathrm{of}\;\mathrm{WT}-\mathrm{FI}\;\mathrm{of}\;\mathrm{eGFP}\right)}$$

where FI represents the fluorescence intensity, V represents the variant, WT refers to wild-type eGFP-LCI, and eGFP serves as the negative control.

### 4.4 Purification

The fusion proteins (eGFP-AP and the negative control eGFP) were expressed in *E. coli* BL21 (DE3) gold cells. Flask expression was carried out as previously described.^42^ The fusion proteins, which included an N-terminal His6-tag, were purified using a prepacked Ni-IDA 2000 column (Macherey-Nagel GmbH & Co. KG, Düren, Germany). Desalting was performed using PD-10 Desalting Columns (Cytiva, Marlborough, MA) according to the manufacturer’s recommended protocol.

### 4.5 Surface Plasmon Resonance

SPR spectroscopy was used to evaluate the surface coverage of eGFP, eGFP-LCI WT, and eGFP-LCI L4H on PS-coated gold sensor chips, with protein concentrations ranging from 50 to 2000 nM. The measurements were conducted using the MP-SPR Navi 420A ILIVES four-channel SPR system (BioNavis Ltd., Tampere, Finland) at a wavelength of 670 nm. To prepare the PS-coated SPR chips, a 0.2% PS solution (in chloroform) was applied to the surface of the pure gold chips. The PS solution (100 μL) was spin-coated at 3780 rpm (63 rps, gear 6) for 1 min using a KLM SC-10 spin coater (Schaefer-Tec, Germany).

Protein solutions in Tris-HCl buffer (50 mM, pH 8.0) were applied onto the PS-coated SPR chip at a flow rate of 20 μL/min for 10 min, followed by 30 min of Tris-HCl buffer (50 mM, pH 8.0) at the same flow rate. The amount of adsorbed protein was determined based on the sensor response in micro refractive index units (μRIU). The change in signal (ΔμRIU), calculated as the difference in baseline before and after protein injection, was converted into surface coverage (ng/cm^2^) using a conversion factor of 0.0518 ng/cm^2^ per μRIU. This conversion factor was experimentally determined by our group based on blood plasma adsorption on bare gold.^86^ The binding data were then fitted into a one-to-one binding model using the GraphPad Prism software (Version 10). This model assumes that a single binding site on the surface interacts with a single ligand molecule, allowing determination of the maximum surface coverage.

### 4.6 MD Simulation

The amorphous PS model was initially generated using the CHARMM-GUI platform.^87^ The CHARMM-GUI polymer builder interface^88^ was utilized to create a disordered, amorphous configuration of PS molecules, suitable for simulating bulk polymer properties. Following the generation of the initial structure, GROMACS (version 2021)^89^ was used for model equilibration. The equilibration process ensured that the system reached a stable state suitable for further simulations. The target density of the amorphous PS model was maintained between 0.96 and 1.05 g/cm^3^, with the final density of the model approximating 0.99 g/cm^3^, which is within the acceptable range for PS (Figure 6).

**Figure 6:**
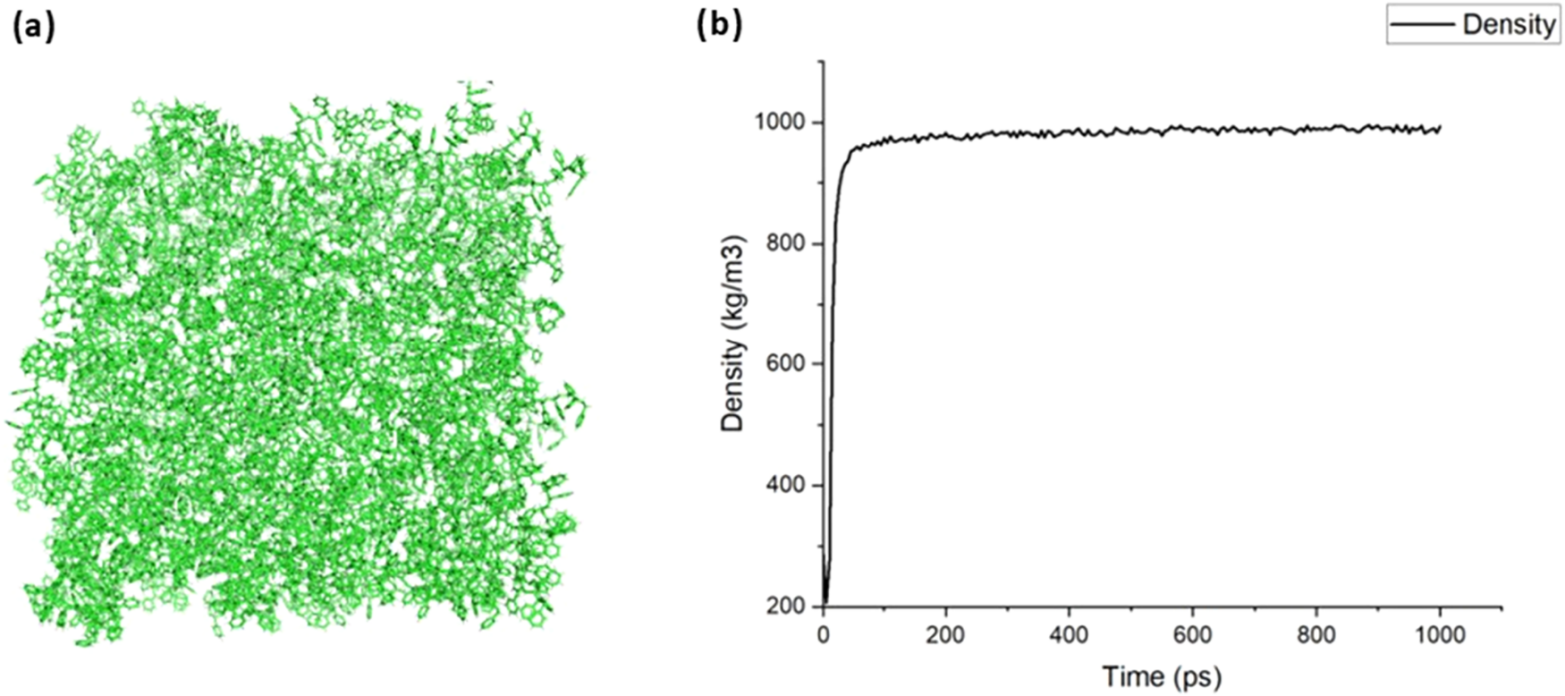
Amorphous PS polymer model. (a) Final structure of the amorphous PS surface. (b) Density profile of the PS system during equilibration.

All simulations were performed by the GROMACS (version 2021) program^89^ with the CHARMM36 force field for both the peptide and polymer.^90^ In the MD simulation of the LCI-PS interaction, an amorphous PS surface was used. The peptide was placed 10 Å above the surface to allow natural, unbiased interactions during the simulation. The system was solvated in a triclinic box (160 × 160 × 150 Å) containing approximately 1,08,257 SPC water molecules. The total system consisted of around 364185 atoms. The system was first energy minimized using the steepest descent algorithm with a maximum force tolerance of 500 kJ/mol/nm over 5000 steps. After energy minimization, the system was equilibrated at 300 K in two stages. First, an NVT ensemble was applied for 100 ps with a 1 fs time step using the V-rescale thermostat to maintain a constant temperature. Following the NVT equilibration, an NPT ensemble was used for 500 ps with pressure coupling controlled by the Parrinello–Rahman barostat to maintain 1 bar pressure, and a time step of 2 fs was used during this phase. Production MD simulations were performed for 100 ns with positional restraints applied to the backbone atoms of the PS layer. Each LCI-PS complex was simulated in three independent runs. Visualization and trajectory analysis were carried out using Visual Molecular Dynamics (VMD, Version 1.9.3)^91^ and PyMOL (The PyMOL Molecular Graphics System, Version 2.5.4 Schrodinger, LLC.).

### 4.7 Binding Free Energy Calculations

The binding free energies of the LCI-WT and the LCI-L4H variant to the PS surface were estimated using the molecular mechanics/Poisson–Boltzmann surface area (MM/PBSA) approach. MD simulations were run for 100 ns, and the last 50 frames (corresponding to ∼10 ns) from each of the three independent runs were analyzed using the *gmx_MMPBSA* tool.^92^

## Supplementary Material



## Notes

Structural representation of the eGFP-LCI fusion protein; overview of the fluorescence-based binding assay; lysate volume optimization; binding assay deviation; comparison of surface coverages of eGFP-LCI variants using SPR; SPR binding profiles of eGFP-LCI WT and L4H with the PS surface; secondary structure of LCI -WT and LCI-L4H in MD simulation system; MD simulation analyses; contact frequency heatmaps for LCI-WT and LCI-L4H variants interacting with PS; CD spectra of eGFP, eGFP-LCI WT, and eGFP-LCI L4H; sequence of LCI-WT and a list of LCI variants with expression levels below saturation (PDF)

## Abbreviations

PS: polystyrene
MBPs: material-binding peptides
LCI: liquid chromatography peak I
SPR: surface plasmon resonance
MD: molecular dynamics
nMBPs: naturally occurring binding peptides
CBMs: carbohydrate-binding modules
eMBPs: engineered MBPs
PET: polyethylene terephthalate
PP: polypropylene
SDM: site-directed mutagenesis
eGFP: enhanced green fluorescent protein
MTP: microtiter plate
CV: coefficient of variation
μRIU: micro refractive index unit
RMSF: root-mean-square fluctuation
RMSD: root-mean-square deviation
Rg: radius of gyration
CD: circular dichroism
