# Supplementary material for: LCI Engineering for Improved Polystyrene Binding: The Impact of Aromatic Amino Acid Substitutions
Source: ACS Omega. 2025 Sep 30;10(40):47227–36. doi: 10.1021/acsomega.5c06026 (PMC12529122; doi:10.1021/acsomega.5c06026)
Supplement: Supplementary file 1 [file ao5c06026_si_001.pdf]

Supporting Information

LCI Engineering for Improved Polystyrene Binding: The Impact of Aromatic Amino Acid Substitutions

Raghda A. Singab‡[a,c], Shuaiqi Meng‡[a], Ulrich Schwaneberg[a,b*]

[a] Lehrstuhl für Biotechnologie, RWTH Aachen University, Worringerweg 3, 52074 Aachen, Germany
[b] DWI-Leibniz Institut für Interaktive Materialien, Forckenbeckstrasse 50, 52074 Aachen, Germany

[c] Microbiology and Immunology Department, Faculty of Pharmacy, Ain Shams University, African Union Organization St., Abbassia, 11566 Cairo, Egypt

*Corresponding author
E-Mail address: u.schwaneberg@biotec.rwth-aachen.de
‡These Authors contributed equally

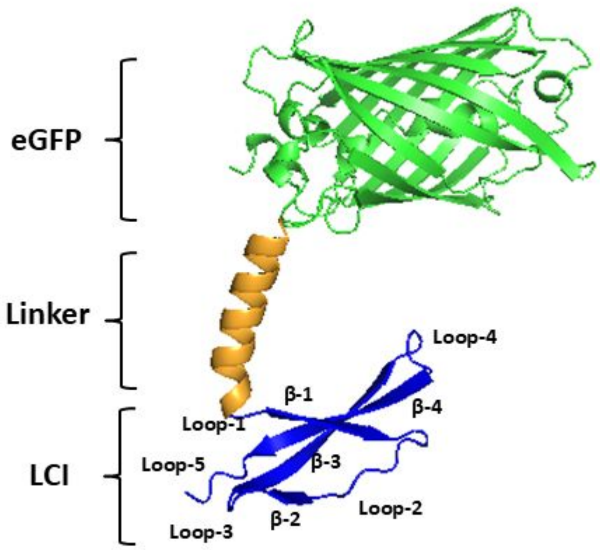

Figure S1. Structural representation of the eGFP-LCI fusion protein. The secondary structure of LCI is labeled, including beta-strands (β-1 to β-4) and loops (Loop-1 to Loop-5). This fusion construct was used for fluorescence-based binding assays to assess interaction with polystyrene surface.

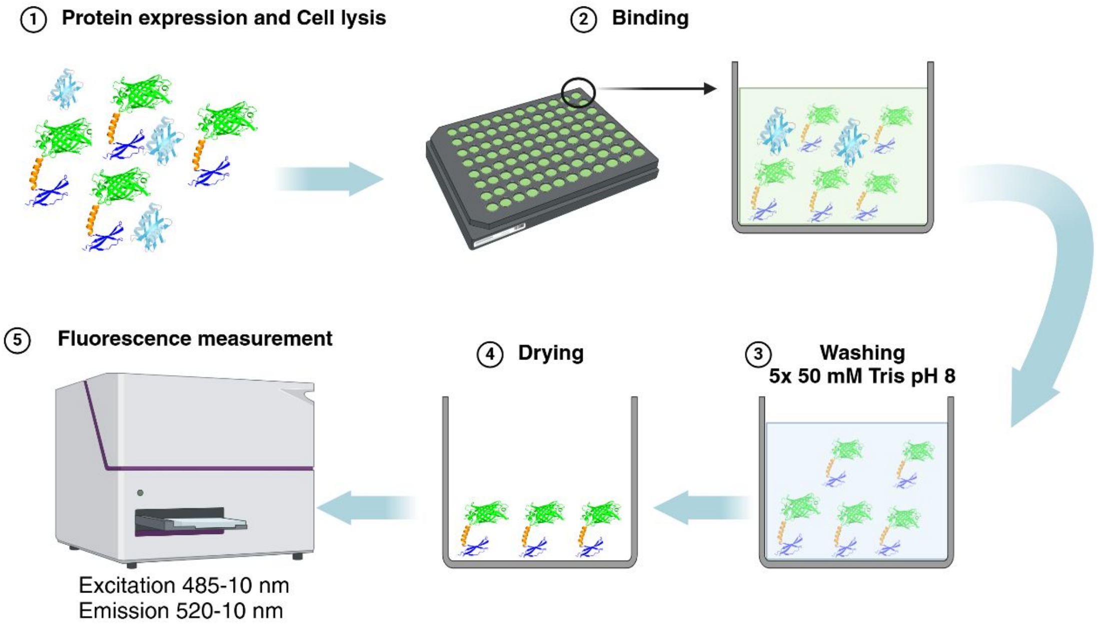

Figure S2. Overview of the fluorescence-based binding assay. (Generated in Biorender: Singab, R. (2025) https://BioRender.com/q42x566)

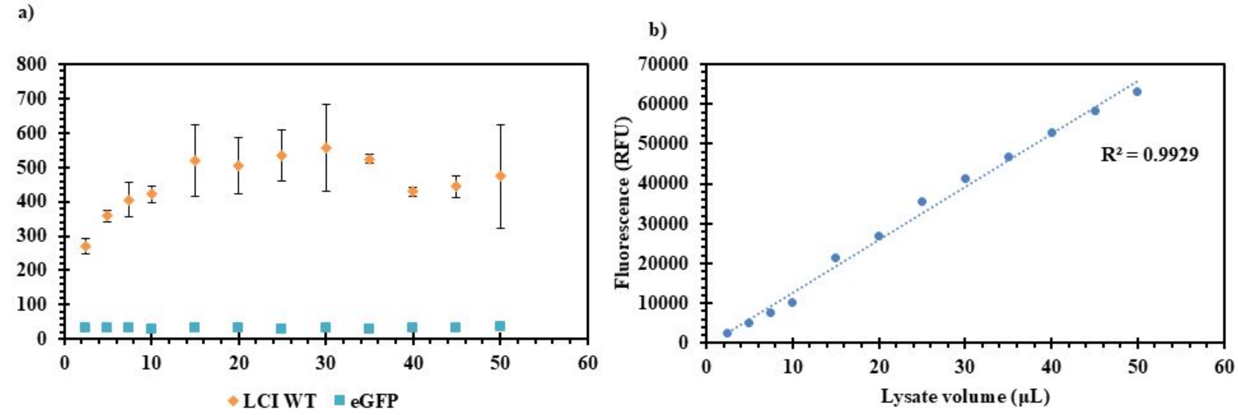

Figure S3. Lysate volume optimization for the binding assay. a) Fluorescence signal measured for varying lysate volumes (2.5–50 µL) containing eGFP-LCI (orange) and eGFP (blue) after washing. b) Linear correlation between the initial fluorescence signal and lysate volume for eGFP-LCI.

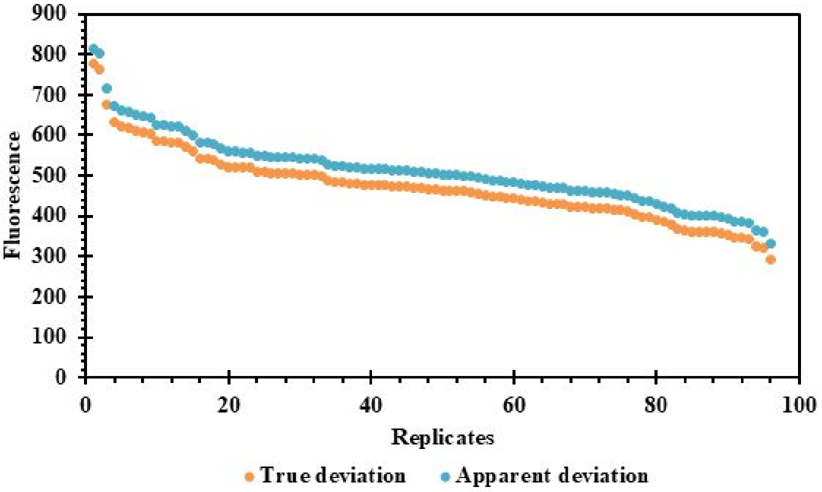

Figure S4. Fluorescence intensity measurements of eGFP-LCI WT binding to PS across 96 replicates. The graph compares the fluorescence values with true standard deviation (orange) and apparent standard deviation (blue) across 96 replicates. The true coefficient of variation (CV) was calculated after subtracting the background fluorescence of eGFP.

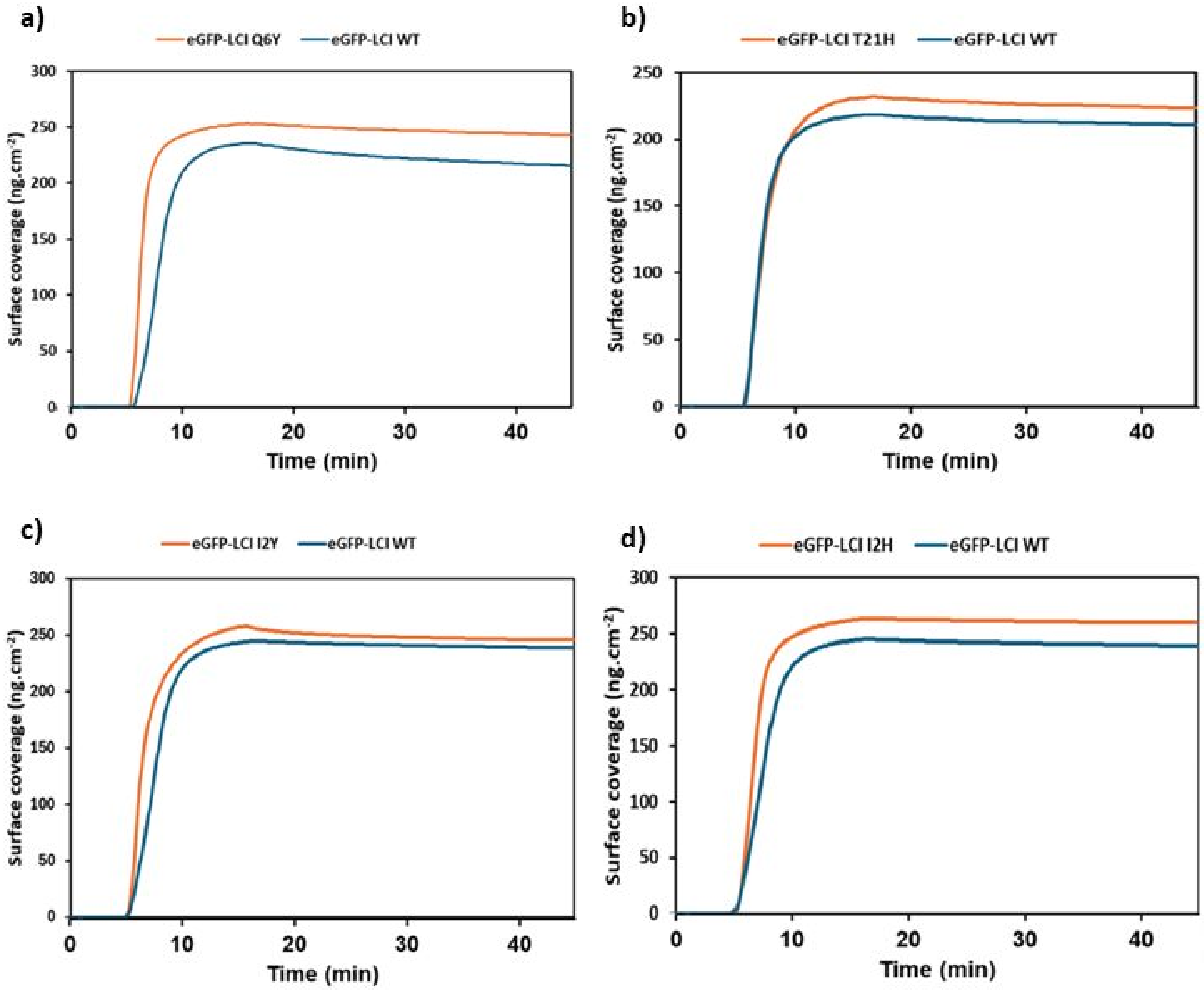

Figure S5. Comparison of surface coverages at 500 nM of LCI variants eGFP-LCI Q6Y (a), eGFP-LCI-T21H (b), eGFP-LCI I2Y (c), and eGFP-LCI I2H (d).

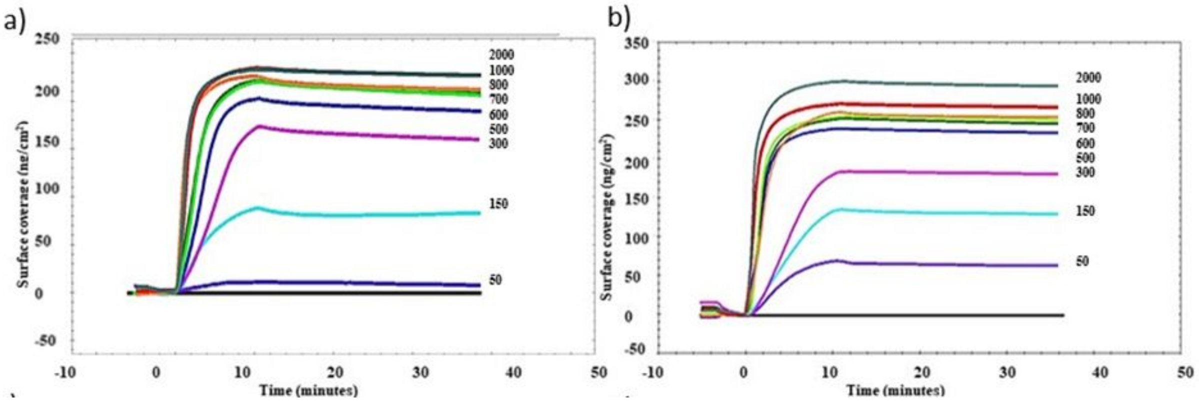

Figure S6. SPR binding profiles showing the interaction of a) eGFP-LCI WT b) eGFP-LCI L4H with the PS surface at concentrations ranging from 50 to 2000 nM. Each curve represents a specific concentration, demonstrating surface coverage over time for both.

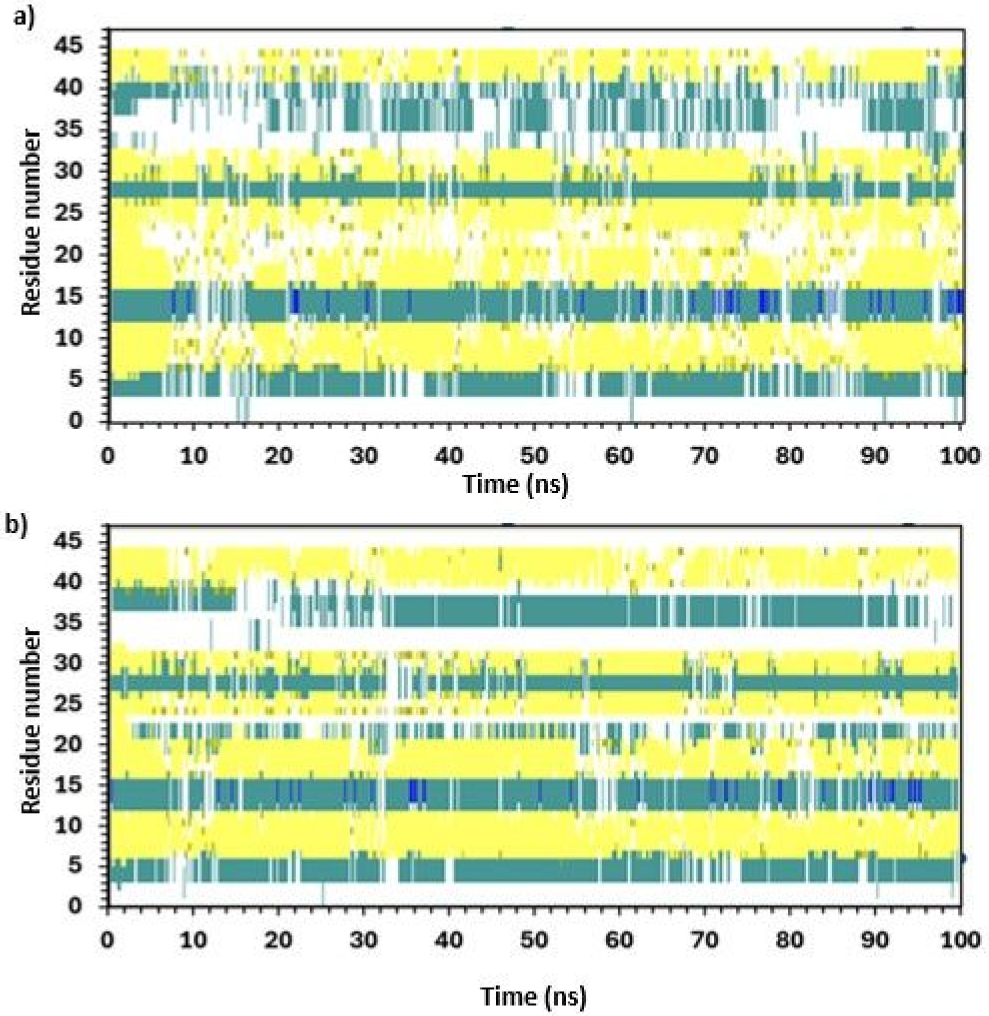

Figure S7. Stability of LCI-WT and LCI-L4H variant secondary structure over 100 ns MD simulation.

(a) The stability of the LCI-WT secondary structure during a 100 ns MD simulation is shown. The y-axis represents the amino acid sequence positions, and the x-axis shows the simulation time. Color-coding highlights different secondary structures: yellow for coil or random structures, green for β-strands, and blue for helices. (b) The stability of the LCI-L4H variant's secondary structure over a 100 ns MD simulation is shown. The same color scheme applies. The variant shows a similar stability pattern to the WT, with consistent β-strand formation and minor fluctuations in coil regions.

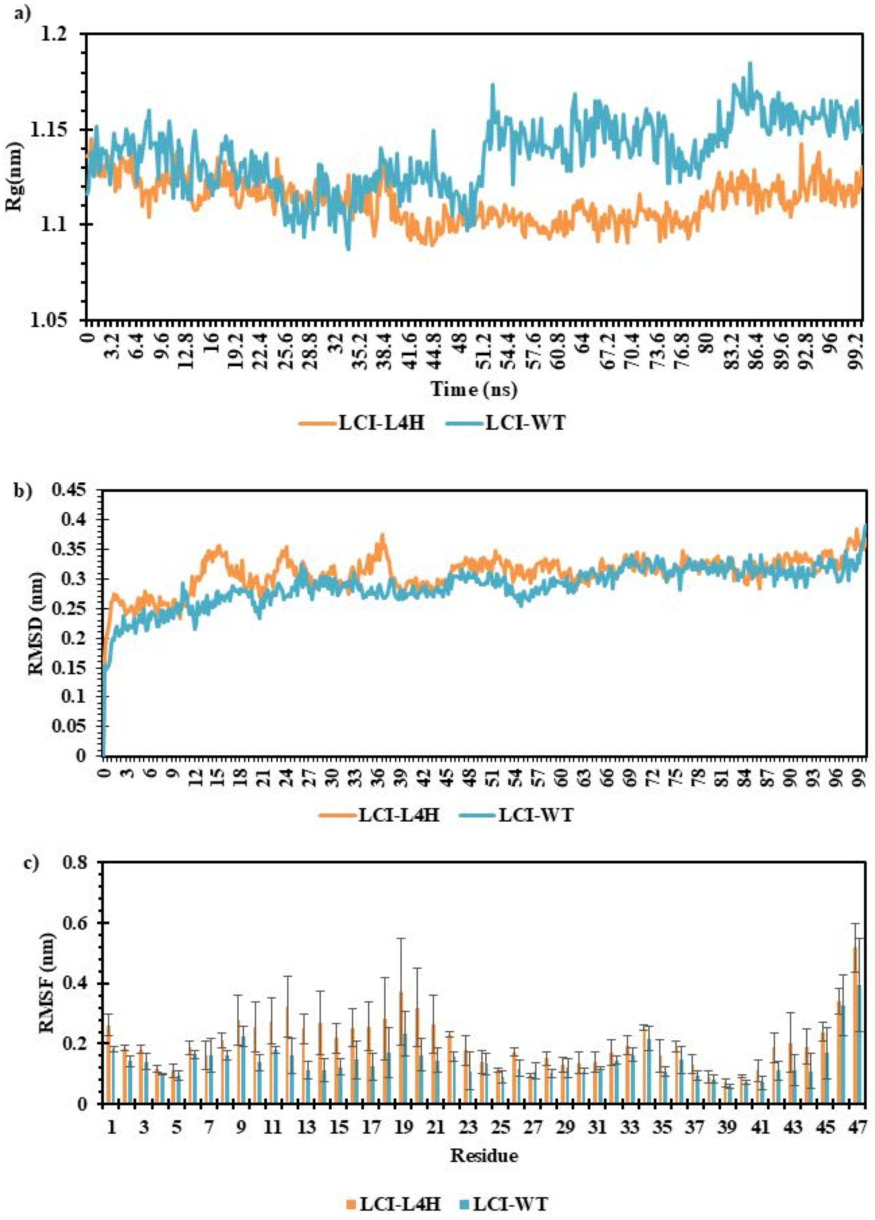

Figure S8. MD simulation analysis of the LCI-WT (blue) and LCI-L4H (orange) variant on PS surface over a 100 ns. a) Rg (in nm) plot for LCI-WT and LCI-L4H variant over simulation time, showing structural compactness of LCI-WT and LCI-L4H. b) RMSD (in nm) plot for LCI-WT and LCI-L4H variant over simulation time, showing the overall deviation in protein conformation during the simulation. c) RMSF (in nm) plot for LCI-WT and LCI-L4H variant over simulation time, quantifying the flexibility of each residue, with higher values indicating greater flexibility. Error bars show the standard deviation for each residue's fluctuation.

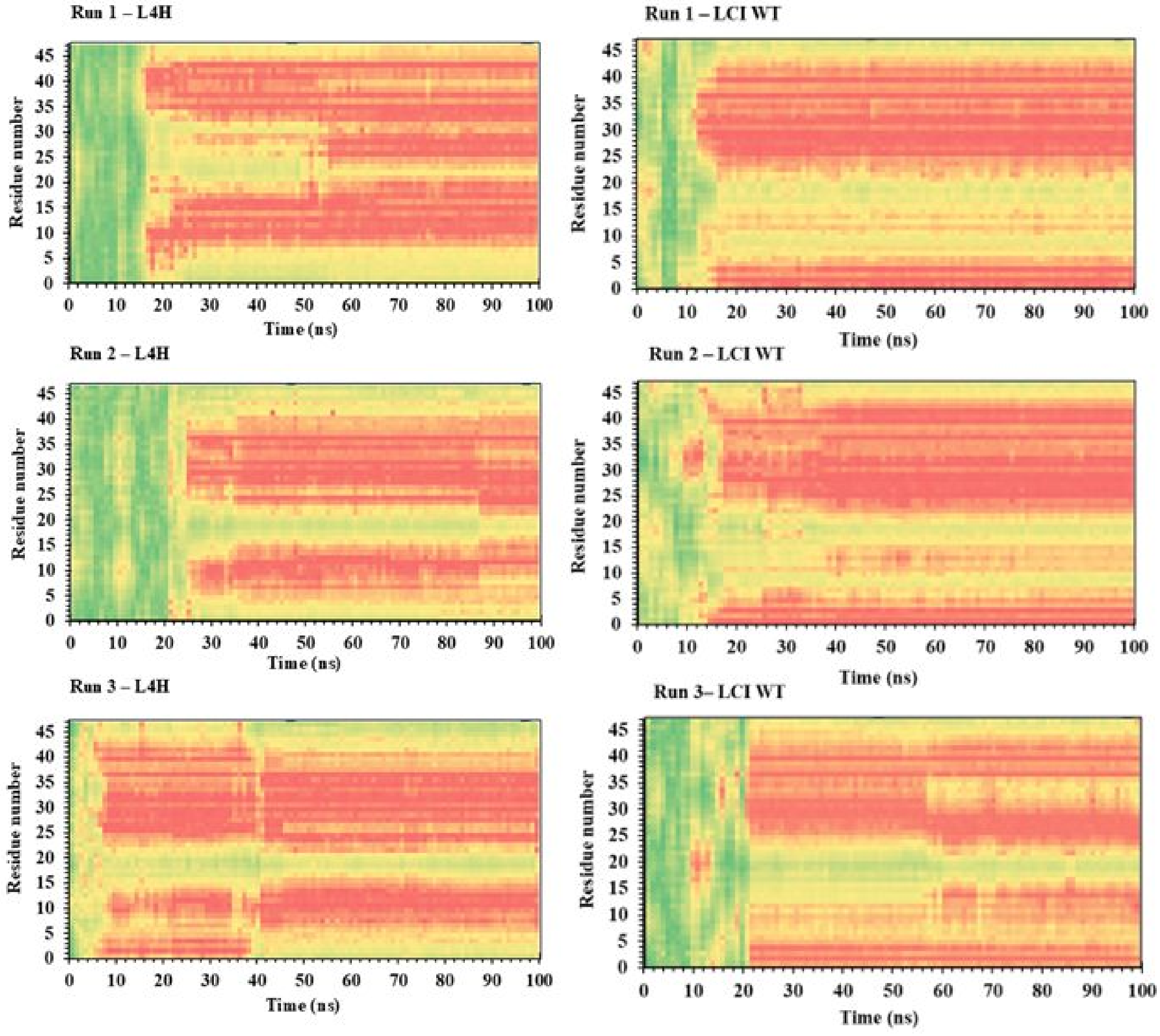

Figure S9. Binding contact frequency for LCI-L4H and LCI-WT variants over 100 ns MD simulations. The heat maps display the contact frequency between PS surface and the LCI-L4H or LCI-WT variant over a 100 ns during three separate MD simulation runs. On the left are the contact maps for the LCI-L4H variant, and on the right are those for the LCI-WT. The x-axis represents time (ns), while the y-axis shows residue numbers.

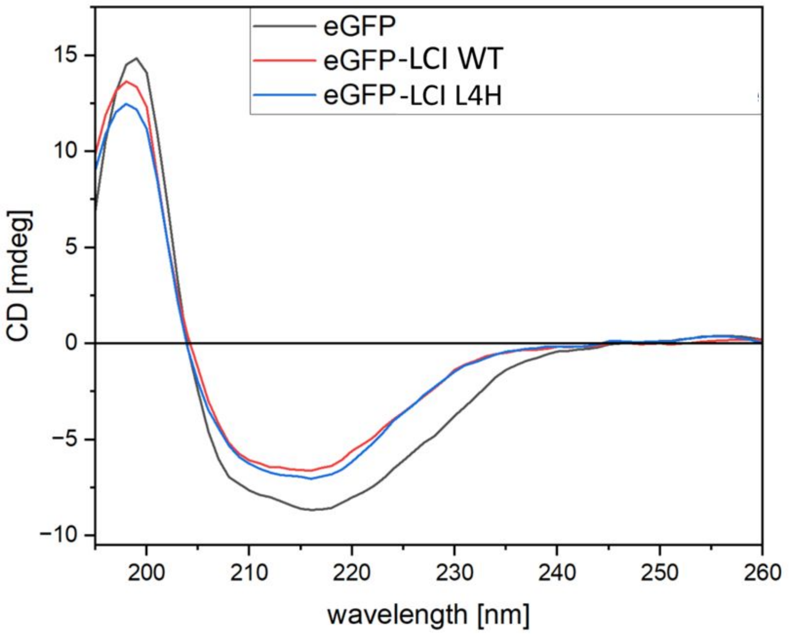

Figure S10. CD spectra of eGFP, eGFP-LCI WT, and eGFP-LCI L4H

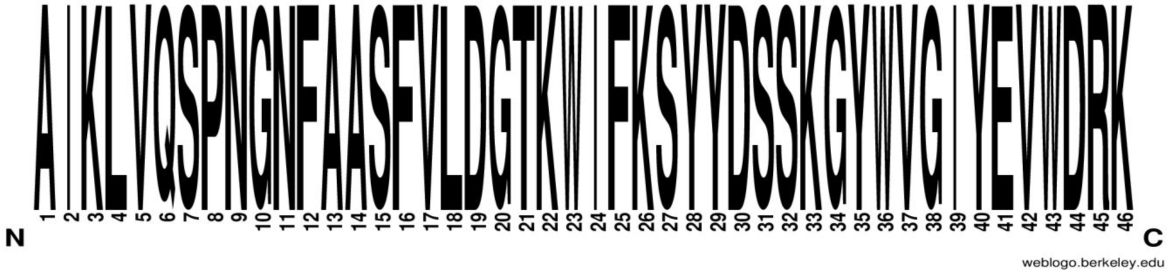

Figure S11. Sequence logo of LCI-WT (generated using WebLogo; weblogo.berkeley.edu).

Table S1. LCI variants showing expression levels below saturation.

Number	Variant
1	A1W
2	N11F
3	S27Y
4	S33W
5	S33Y
6	K34H

7	G35Y
8	G35F
9	G39Y
10	G39W
11	G39F
12	I40H
